# Retraction: Tim-3 Expression in Cervical Cancer Promotes Tumor Metastasis

**DOI:** 10.1371/journal.pone.0308113

**Published:** 2024-07-25

**Authors:** The PLOS ONE Editors

Following the publication of this article [1], concerns were raised that the Fig 5D ADV-GFP panel partially overlaps with the Fig 5D PBS panel and the Fig 5D ADV-AsTim-3 panel. A Correction [2] which replaced the Fig 5D ADV-GFP panel was previously issued. However, this Correction [2] did not resolve the concern that the Fig 5D PBS panel and the Fig 5D ADV-AsTim-3 also appear to be part of the same transwell invasion assay membrane, despite these panels being used to represent different experimental conditions.

In response to the remaining concerns with Fig 5, the first author provided underlying image data from the original experiments for the results presented in Figs 5A and 5D. Upon editorial review of these data, PLOS noted additional image similarity concerns.

The first author stated that the concerns with the underlying image data provided were due to errors in image file labeling and selection.

In light of the above concerns that raise questions regarding the overall data handling and the integrity of the original data underlying Fig 5, the *PLOS ONE* Editors retract this article.

YC responded but expressed neither agreement nor disagreement with the editorial decision. XZ, XH, QL, LG, LJ, MH, and JZ either did not respond directly or could not be reached.
